# A Rabbit Model for the Evaluation of Drugs for Treating the Chronic Phase of Botulism

**DOI:** 10.3390/toxins13100679

**Published:** 2021-09-24

**Authors:** Amram Torgeman, Eran Diamant, Eyal Dor, Arieh Schwartz, Tzadok Baruchi, Alon Ben David, Ran Zichel

**Affiliations:** Department of Biotechnology, Israel Institute for Biological Research, Ness Ziona 7410001, Israel; amit@iibr.gov.il (A.T.); erand@iibr.gov.il (E.D.); eyalo@iibr.gov.il (E.D.); ariehs@iibr.gov.il (A.S.); tzadokb@iibr.gov.il (T.B.); alonb@iibr.gov.il (A.B.D.)

**Keywords:** botulinum, chronic, sublethal, antitoxin, animal model

## Abstract

Antitoxin, the only licensed drug therapy for botulism, neutralizes circulating botulinum neurotoxin (BoNT). However, antitoxin is no longer effective when a critical amount of BoNT has already entered its target nerve cells. The outcome is a chronic phase of botulism that is characterized by prolonged paralysis. In this stage, blocking toxin activity within cells by next-generation intraneuronal anti-botulinum drugs (INABDs) may shorten the chronic phase of the disease and accelerate recovery. However, there is a lack of adequate animal models that simulate the chronic phase of botulism for evaluating the efficacy of INABDs. Herein, we report the development of a rabbit model for the chronic phase of botulism, induced by intoxication with a sublethal dose of BoNT. Spirometry monitoring enabled us to detect deviations from normal respiration and to quantitatively define the time to symptom onset and disease duration. A 0.85 rabbit intramuscular median lethal dose of BoNT/A elicited the most consistent and prolonged disease duration (mean = 11.8 days, relative standard deviation = 27.9%) that still enabled spontaneous recovery. Post-exposure treatment with antitoxin at various time points significantly shortened the disease duration, providing a proof of concept that the new model is adequate for evaluating novel therapeutics for botulism.

## 1. Introduction

Botulinum neurotoxins (BoNTs), produced by *Clostridium botulinum* strains, are the most potent toxins in nature, with an estimated human median lethal dose (HLD_50_) of 1 ng/kg body weight [1,2]. There are at least seven serotypes of BoNTs (A-G), of which serotypes A, B, E, and rarely F are responsible for most cases of human botulism [3]. Botulinum toxins are synthesized as large protein complexes, consisting of a neurotoxin, nontoxic nonhemaglutinin (NTNH), and in some serotypes nontoxic hemaglutinins (HA) [4]. The active form of the neurotoxin consists of 100 (heavy chain) and 50 (light chain) kDa polypeptide chains, which are joined by a disulfide bridge [5]. The C-terminal half (∼50 kDa) of the heavy chain (Hc) is the receptor binding domain, while the N-terminal half (Hn) is the translocation domain of the neurotoxin. The catalytic domain is a zinc-endopeptidase confined to the light chain (LC) [6].

BoNTs exert their action on the cholinergic system at presynaptic motor-neuron terminals by cleaving SNARE proteins, thus blocking acetylcholine transmission across neuromuscular junctions. As a result, symmetrical descending flaccid paralysis occurs, affecting the cranial nerves and eventually the diaphragm and accessory breathing muscles [7]. Widespread outbreaks of food-borne botulism might have severe implications, since patients without adequate treatment may die [8,9,10]. In addition, due to their extreme potency, BoNTs are classified as category A biothreat agents [11].

Currently, the only licensed drug therapy for botulism is an immunoglobulin-based antitoxin, which mostly neutralizes circulating BoNT molecules that have not yet entered neurons [12]. Therefore, at a certain time point, after a critical amount of BoNT molecules are already within neurons, the efficacy of the antitoxin treatment dramatically declines. Indeed, data from studies conducted on animals and from human clinical cases support the notion that there is a critical “therapeutic time window” for effective antitoxin treatment [8,13,14,15,16,17]. In cases where antitoxin is no longer effective, hospitalization under supportive intensive care and mechanical ventilation may be required. This clinical condition, here termed the “chronic” phase of botulism, is characterized by prolonged paralysis and breathing difficulties that may last for weeks and even months until complete recovery is achieved [18]. In the chronic phase, when antitoxin is irrelevant, next-generation intraneuronal anti-botulinum drugs (INABDs) designed to block the LC inside the cells are required. Such therapeutics may halt the progression of intoxication and accelerate the recovery of paralyzed muscles.

The vast majority of animal models of botulism are based on exposure to lethal toxin doses, resulting in an acute fatal disease characterized by a rapid deterioration within hours or a few days, depending on the toxin dose and serotype. Consequently, they may lack the appropriate resolution and sensitivity required to test the efficacy of potential INABDs that are intended to act during the chronic phase of the disease. Therefore, as an alternative to lethal models, we reasoned that intoxication with a sublethal amount of BoNT would generate prolonged disease, reflecting the chronic phase of botulism. This new approach may be more appropriate for the evaluation of INABDs that can potentially accelerate recovery.

The development of an animal model for the chronic phase of botulism needs to rely on the onset of, and recovery from, defined clinical symptoms. To achieve this requirement, it is essential to establish an objective and quantitative botulism symptom parameter. In this respect, we recently reported the development of a spirometry-based rabbit model of botulism and used it to evaluate antitoxin efficacy after the manifestation of respiratory symptoms in animals exposed to lethal doses of BoNT/A, BoNT/B, and BoNT/E [19,20]. In this model, a relevant respiratory symptom was established, based on the measurement of minute volume (MV) (the volume of air inhaled per minute). To the best of our knowledge, this spirometry model is the first animal model able to simulate a beneficial antitoxin treatment after the onset of objective and quantitative clinical symptoms of botulism. Importantly, the model exhibits a high correlation with human botulism, both in terms of the pathophysiology of the disease, and in the differential serotypic pattern of antitoxin treatment efficacy. These observations prompted us in the present study to apply rabbit spirometry principles for the development of a novel model of chronic botulism and spontaneous recovery.

## 2. Results

### 2.1. Determining the Optimal Sublethal BoNT/A Dose to Induce Chronic Botulism

**Figure 1:**
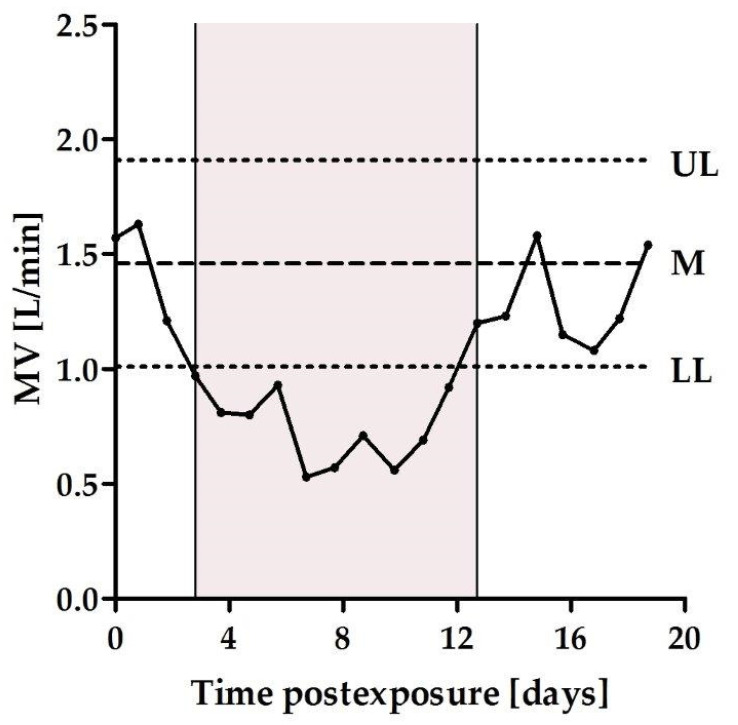
Respiration profile of a rabbit exposed to a sublethal dose of BoNT/A. Representative MV data, as a function of time post-exposure from a rabbit exposed to 0.85 RbIMLD_50_ of BoNT/A. The mean MV (M) of normal breathing is denoted by a dashed line. The upper (UL) and lower (LL) confidence limits (mean ± 2 × SD) are represented by dotted lines. The zero time indicates the time of toxin administration. The highlighted area represents the disease duration between the TTS and the TTR (left and right vertical lines, respectively).

In our previous studies, respiration distress, reflected by the MV parameter, following lethal exposure to BoNT types A, B, and E, was used to quantify early symptoms of acute botulism and served as a trigger for treatment [19,20]. The goal of the current study was to use the MV parameter to establish a model of the chronic phase of type A botulism. To this end, respiratory deviations were measured subsequently to intoxication with 0.5, 0.65, 0.75, and 0.85 intramuscular rabbit LD_50_ (RbIMLD_50_) of BoNT/A. Normal MV values, obtained individually by daily measurements prior to intoxication, were used to determine the lower and upper confidence limits. Following intoxication, animals reaching MV values below their lower confidence limit were considered symptomatic [19,20]. The frequency of symptomatic rabbits in each dosing group was increased in a dose-dependent manner: 50%, 75%, and 80% with toxin doses of 0.5, 0.65, and 0.75 RbIMLD_50_, respectively (Table 1). One hundred percent of the rabbits became symptomatic only in the 0.85 RbIMLD_50_ exposed group (Table 1). Notably, all of the rabbits survived when administered 0.85 RbIMLD_50_, despite its proximity to a lethal exposure dose. The intoxication with 0.85 RbIMLD_50_ elicited the most consistent and prolonged symptomatic phase, which lasted ~12 days on average, from symptom onset until spontaneous recovery (Figure 1). Therefore, the dose of 0.85 RbIMLD_50_ of BoNT/A was selected to induce chronic botulism. Evidently, these observations emphasize the importance of the fine-tuning process needed to determine the exact sublethal dose for establishing this model.

### 2.2. Characterization of the Chronic Phase of Botulism Induced by a Sublethal Dose of BoNT/A

Spirometry data (MV values) of each of the thirteen rabbits exposed to a BoNT/A sublethal dose of 0.85 RbIMLD_50_ were further analyzed to characterize the spirometry profile and define the quantitative disease parameters (Figure 1). The parameters included the individual time to symptoms (TTS) and time to recovery (TTR), and the difference between them was referred to as the disease duration, i.e., the symptomatic chronic phase. TTS was defined as the time post-exposure at which a statistically significant deviation (mean MV minus 2 × standard deviation (SD)) from the pre-exposure limit had occurred.

**Figure 2:**
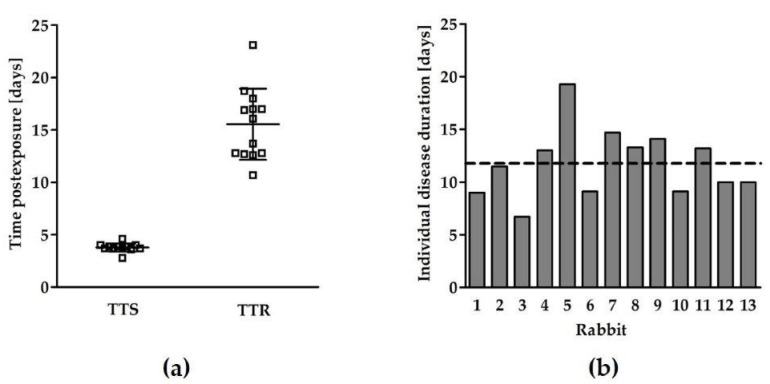
Analysis of the chronic phase of botulism following sublethal intoxication. Rabbits (*n* = 13) were exposed to 0.85 RbIMLD_50_ of BoNT/A, and the MV values were obtained by daily spirometry monitoring. (**a**) TTS and TTR were individually determined based on the MV parameter. (**b**) Disease duration of each rabbit was calculated by subtracting its respective TTS from TTR. The dashed line denotes the mean disease duration.

The individual TTS values were highly reproducible, with a mean of 3.8 days and a relative standard deviation (RSD) of 10.8% (Figure 2a). This parameter can allow us to assess the beneficial effects of anti-BoNT compounds either by the delay in symptom onset or by the complete prevention of symptom manifestation. The individual TTR values (spontaneous recovery without treatment) were also consistent, with a mean of 15.5 days and an RSD of 21.9% (Figure 2a). Accordingly, the mean disease duration was determined (11.8 days, RSD = 27.9%, Figure 2b). This relatively prolonged symptomatic phase may enable a reliable assessment of the efficacy of anti-BoNT/A compounds during the chronic phase of botulinum intoxication.

### 2.3. Qualification of the Model Using Botulinum Antitoxin

**Figure 3:**
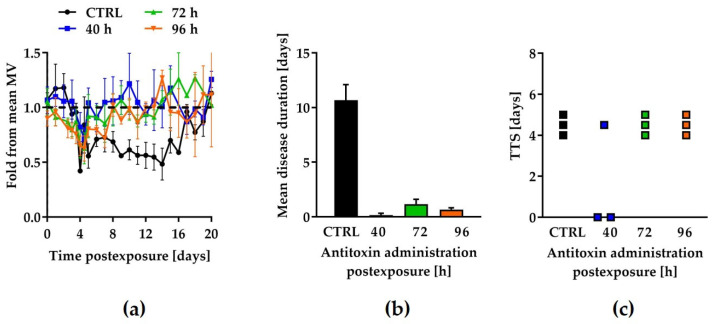
Postintoxication treatment with antitoxin shortens the disease duration. Rabbits (*n* = 12) were exposed to 0.85 RbIMLD_50_ of BoNT/A without (black, *n* = 3) and with antitoxin treatment (215 IU/kg) either 40 h (blue, *n* = 3), 72 h (green, *n* = 3), or 96 h (orange, *n* = 3) after intoxication, and the TTS and disease duration were then determined and compared. (**a**) Inhalation profile during the disease course of the control and the antitoxin-treated rabbits (respiration was monitored daily). The dashed line represents the baseline normalized mean MV value, expressed as the fold change relative to the mean MV. (**b**) TTS was determined for each rabbit based on the decreased MV parameter. Each square refers to an individual animal. (**c**) Mean disease duration values of the antitoxin-treated rabbits at various time points.

Pharmaceutical antitoxin was used as a means to validate the chronic model by testing its beneficial effects with respect to TTS and the disease duration. To this end, twelve rabbits were exposed to 0.85 RbIMLD_50_ of BoNT/A and divided into four groups (*n* = 3), treated with 215 IU/kg of antitoxin at different time points post-exposure: (1) 40 h, (2) 72 h, (3) 96 h, and (4) control (phosphate buffered saline) (Figure 3a). Of note, the three rabbits in the 96-h treatment group received antitoxin immediately after respiratory symptoms manifested. The TTS and disease duration for the control rabbits were 4.5 and 10.7 days, respectively (Figure 3b,c). Importantly, two out of three rabbits in the earliest (40 h) post-exposure group were completely nonsymptomatic, while the third rabbit had a TTS of 4.5 days and a disease duration of 0.5 days (the mean disease duration of this group was 0.17 days). Thus, the beneficial effect of antitoxin treatment at 40 h post-exposure eliminated symptom onset or significantly shortened the disease duration. An identical TTS (4.5 days) was obtained in the 72-h and 96-h groups, and their mean disease duration was significantly shortened to 1.2 and 0.7 days, respectively. The reproducibility of the TTS data, together with the demonstration of antitoxin efficacy, substantiates the applicability of this system to serve as a model of chronic botulism for testing new anti-BoNT/A drug candidates.

## 3. Discussion

INABDs have the potential to provide a useful treatment during the chronic phase of botulism, when antitoxin is ineffective. However, most of the current animal models available for botulism are based on the induction of short-term, acute botulism by lethal intoxication and thus cannot be used for testing INABDs in the chronic phase of the disease. Moreover, intoxication with lethal doses may pose an extreme challenge for a potentially effective INABD. Moderate intoxication may provide a sensitive model that will allow for the detection of a beneficial therapeutic effect from INABDs. Therefore, animal models in which a chronic phase of botulism is induced are required.

To date, only a few attempts have been made to develop models for chronic botulism [16,21]. Analogous to severe cases of human botulism, simulating the chronic phase of the disease in animals can theoretically be achieved by extending the survival of intoxicated animals by mechanical ventilation following lethal exposure to BoNT. However, this approach is highly challenging to conduct. Alternatively, a chronic phase can be directly induced by intoxication with a sublethal amount of BoNT. Most recently, our research group developed a running wheel system for detecting botulism symptoms in mice based on voluntary running. Exposure to a sublethal dose of BoNT/A (0.3 LD_50_) induced a substantial chronic phase (a disease duration of 16–18 days) [16]. In another recent study of mice exposed to sublethal toxin doses, chronic botulism for BoNT/A, BoNT/B, and BoNT/E was developed based on digit abduction scores and moderate-to-severe abdominal paradoxical breathing measurements [21].

In the current report, a model for the chronic phase of botulism was developed for the first time in rabbits. In accordance with the Animal Rule of the U.S. Food and Drug Administration [22], the development of a rabbit model expands the evaluation toolkit of botulism therapeutics. Notably, rabbits are the third most commonly used species for experimental research in the European Union [23]. Moreover, rabbits are larger than mice, and it is easier to apply various procedures, such as blood sampling and intravenous administration of drugs, to rabbits than to mice [24]. Rabbits are phylogenetically closer to humans than rodents, and due to their anatomical, physiological, genetic, and biochemical similarities to humans, rabbits are used as animal models for human diseases in various medical research fields [25,26].

The model described in the current work relies on the definition of an objective and quantitative human-related clinical symptom of botulism in rabbits, expressed as the deviation from normal MV values following sublethal intoxication with 0.85 RbIMLD_50_. Measuring this clinical symptom allows for the exact determination of two fundamental characteristics of the disease: TTS and disease duration. The induced disease is characterized by a prolonged symptomatic period that is followed by spontaneous recovery.

To date, only a few publications have shown beneficial treatment of botulism with experimental INABDs [21,27,28,29,30,31,32,33,34]. Due to the absence of approved INABDs, the proof of concept of our model, in terms of measuring the beneficial effects of potential anti-BoNT/A compounds, was assessed by pharmaceutical antitoxin. In all rabbits exposed to a sublethal dose of BoNT/A, treatment with antitoxin 40 h, 72 h, and 96 h (the last being immediately administered after symptom onset) after toxin exposure either completely prevented respiratory symptoms or significantly shortened the symptomatic phase of botulism compared to the control groups. Interestingly, TTS was not delayed among symptomatic rabbits that were treated 40 h and 72 h post-exposure. Collectively, these results validate the new model as a reproducible platform for testing the efficacy of anti-botulinum drugs in halting disease progression during the chronic phase.

Additionally, the data can give various insights into the neutralizing mechanism of the circulating toxin by antitoxin. Evidently, two out of three rabbits did not become symptomatic when treated 40 h post-exposure, while symptoms were detected in all animals treated 72 h post-exposure. This implies that at a certain time point, between 40 h and 72 h post-exposure, sufficient intracellular toxin has accumulated to generate respiratory symptoms. Notably, the observation that the TTS was not delayed in the antitoxin-treated rabbits may indicate that the innervation of the breathing muscles had already been affected before this time by the amount of toxin that was present intracellularly. A reasonable explanation for the shortening of the disease duration could be related to the neutralization of unbound toxin in the blood. Accordingly, although circulating antitoxin is incapable of neutralizing intracellular toxin, it can prevent the escalation of the disease by neutralizing the residual circulating toxin that would otherwise intoxicate more target cells.

An important aspect of some INABDs, such as small molecule inhibitors, and which can affect the outcome, is their poor pharmacokinetics, as compared to antitoxin [30,35,36,37,38,39]. Hence, either continuous infusion or multiple administrations of the drug may be required to achieve full effectiveness. Of note, repeated administrations and well-established means for the continuous infusion of drugs are prevalent in the clinic. Therefore, it is important to also be able to apply a similar continuous infusion of drugs in animal models. In this respect, we recently developed a new model of prolonged continuous IV infusion in free-roaming rabbits by simple catheterization of a peripherally inserted central catheter (PICC) from the marginal ear vein to the superior vena cava and by using carried minipumps [40]. The combination of the PICC-based infusion model with the chronic botulism model described in the current study can facilitate the evaluation of potential intracellular BoNT inhibitors.

In summary, a new rabbit model of chronic botulism and spontaneous recovery, based on respiratory symptoms, was developed. This model allowed us to demonstrate the prevention of symptom onset and the shortening of disease duration by a well-known effective drug. This model can be used as a platform to test other potential anti-BoNT compounds, especially in the late stages of the disease when antitoxin is no longer effective.

## 4. Materials and Methods

### 4.1. Rabbits

Healthy New Zealand White rabbits, provided by Charles River (Lyon, France), were used in this study. All rabbits were females weighing between 2.5 and 3.5 kg. All experiments were approved by the Israel Institute for Biological Research (IIBR) Animal Care and Use Committee (Ness Ziona, Israel) and were conducted in accordance with the guidelines for the care and use of laboratory animals published by the Israeli Ministry of Health (protocols # RB-11-14 and RB-21-15). All efforts were made to minimize animal suffering. All animals were observed for morbidity and mortality, overt signs of toxicity, and any signs of distress throughout the study.

### 4.2. Bacteria and Toxins

*Clostridium botulinum* type A was obtained from the Israel Institute for Biological Research (IIBR) collection (A198) (Ness Ziona, Israel). A sequence analysis revealed compliance of the neurotoxin genes with serotypes 62A (GenBank accession number M30196) of *C. botulinum* type A1 [41,42]. The toxin complex was prepared from a concentrated supernatant of cultures grown for 6 days in anaerobic conditions. The toxin stock was kept in 50 mM citrate buffer (pH 5.5) at −70 °C. Toxin potency was determined by mouse lethality assay, as previously described [21]. A dose of 1 RbIMLD_50_ was found to be equivalent to 4 mouse intraperitoneal LD_50_ per kg [21].

### 4.3. Antitoxin

Hyperimmune plasma was collected from horses immunized with a toxoid preparation that was obtained by dialyzing the toxin complex against 0.14% formalin at 35 °C for 2 weeks [43]. The fragment crystallizable (Fc) region was removed by pepsin digestion [44], and the neutralizing activity of the purified F(ab’)2 antitoxin was determined according to the European Pharmacopeia [45]. In summary, serial 1.2-fold dilutions of each antitoxin preparation were prepared. Simultaneously, a standard antitoxin preparation (calibrated according to the World Health Organization international standard antitoxin) was diluted to final concentrations of 0.08, 0.10, 0.12, and 0.14 IU/mL. All antitoxin dilutions were then mixed with a fixed toxin test dose, and the mixtures were incubated for 1 h at 25 °C. One milliliter per mouse of each mixture was injected intraperitoneally into four CD-1 mice (Charles River, Margate, Kent, UK), and survival was monitored for 4 days. The antitoxin potency was calculated based on the lowest dilution of antitoxin that failed to protect the animals, as compared with that of the standard antitoxin.

### 4.4. Spirometry

The computer-controlled spirometry system has been previously described [19,20]. A thermal mass flowmeter (model 4100, TSI Inc., Shoreview, MN, USA) was connected to a snout-only mask via the inhalation port of the valve. During measurements, the nonanesthetized rabbits respired freely. Inhalation data were collected at 20 ms intervals (500 data points in 10 s). MV values were analyzed using Microsoft Excel (2013). At least 12 independent measurements, collected individually over 12 days before toxin exposure, were used to calculate the mean and standard deviation (SD) of the MV values for each rabbit. Confidence limits were determined as the mean ±2 × SD [19]. Values below the lower limit in the BoNT-exposed rabbits were attributed to breathing distress owing to intoxication and thus were considered clinical symptoms of botulism.

### 4.5. Antitoxin Efficacy Studies

Following acclimation and individual calculation of the MV-based confidence limit, the rabbits were sublethally intoxicated by injection of 0.5 mL of the indicated BoNT/A dose in gelatin-phosphate buffer (50 mM Na-phosphate, 0.2% gelatin, pH 6.5) into the quadriceps musculature of the hindlimb. Rabbit spirometry was monitored daily post-intoxication. Antitoxin was intravenously (IV) administered to rabbits at the indicated time post-exposure. The antitoxin dose was equivalent in body weight to the human indicated dose (215 IU/kg). All experiments included rabbits that served as a negative control (toxin only). Recovery was defined either as the time point at which the MV values entered the confidence limit and remained between the limits for the rest of the measurements or when the MV values exceeded 90% of the lower limit of confidence, while the relative standard deviation (RSD) of four successive measurements (including the one from the first criterion) remained at less than 20%.

## Figures and Tables

**Table 1:** Respiratory symptom onset in intoxicated rabbits after exposure to different BoNT/A doses.

BoNT/A Dose (RbIMLD_50_) ^1^	% Symptomatic Rabbits (*n*)
0.5	50 (2)
0.65	75 (4)
0.75	80 (5)
0.85	100 (13)

^1^ Rabbits were exposed to the indicated doses of BoNT/A, and respiratory symptoms were determined for each rabbit based on the MV parameter.

## Data Availability

The data presented in this study are available on request from the corresponding author.

## References

[1] Arnon S.S., Schechter R., Inglesby T.V., Henderson D.A., Bartlett J.G., Ascher M.S., Eitzen E., Fine A.D., Hauer J., Layton M. (2001). Botulinum toxin as a biological weapon: Medical and public health management. J. Am. Med. Assoc..

[2] Gill D.M. (1982). Bacterial toxins: A table of lethal amounts. Microbiol. Rev..

[3] Atassi M.Z., Oshima M. (1999). Structure, activity, and immune (T and B cell) recognition of botulinum neurotoxins. Crit. Rev. Immunol..

[4] Sugiyama H. (1980). *Clostridium botulinum* neurotoxin. Microbiol. Rev..

[5] Pirazzini M., Rossetto O., Eleopra R., Montecucco C. (2017). Botulinum Neurotoxins: Biology, Pharmacology, and Toxicology. Pharmacol. Rev..

[6] Lacy D.B., Tepp W., Cohen A.C., DasGupta B.R., Stevens R.C. (1998). Crystal structure of botulinum neurotoxin type A and implications for toxicity. Nat. Struct. Biol..

[7] Dembek Z.F., Smith L.A., Rusnak J.M. (2007). Botulism: Cause, effects, diagnosis, clinical and laboratory identification, and treatment modalities. Disaster Med. Public Health Prep..

[8] Kongsaengdao S., Samintarapanya K., Rusmeechan S., Wongsa A., Pothirat C., Permpikul C., Pongpakdee S., Puavilai W., Kateruttanakul P., Phengtham U. (2006). An outbreak of botulism in Thailand: Clinical manifestations and management of severe respiratory failure. Clin. Infect. Dis..

[9] Weber J.T., Hibbs R.G., Darwish A., Mishu B., Corwin A.L., Rakha M., Hatheway C.L., El Sharkawy S., El-Rahim S.A., Al-Hamd M.F.S. (1993). A massive outbreak of type E botulism associated with traditional salted fish in Cairo. J. Infect. Dis..

[10] McCarty C.L., Angelo K., Beer K.D., Cibulskas-White K., Quinn K., de Fijter S., Bokanyi R., St Germain E., Baransi K., Barlow K. (2015). Large Outbreak of Botulism Associated with a Church Potluck Meal-Ohio, 2015. MMWR Morb. Mortal. Wkly. Rep..

[11] Centers for Disease Control and Prevention Bioterrorism Agents/Diseases. https://emergency.cdc.gov/agent/agentlist-category.asp.

[12] Sobel J. (2005). Botulism. Clin. Infect. Dis..

[13] Iida H., Ono T., Karashimada T., Ando Y. (1970). Studies on the serum therapy of type E botulism: Absorption of toxin from the gastrointestinal tract. Jpn. J. Med. Sci. Biol..

[14] Kodihalli S., Emanuel A., Takla T., Hua Y., Hobbs C., LeClaire R., O’Donnell D.C. (2017). Therapeutic efficacy of equine botulism antitoxin in Rhesus macaques. PLoS ONE.

[15] Mottate K., Yokote H., Mori S., Horita A., Miyatsu Y., Torii Y., Kozaki S., Iwaki M., Takahashi M., Ginnaga A. (2016). Retrospective survey to evaluate the safety and efficacy of Japanese botulinum antitoxin therapy in Japan. Toxicon.

[16] Schwartz A., Ben David A., Hotoveli M., Dor E., Diamant E., Vivyorka A., Rosen O., Torgeman A., Zichel R. (2021). A Novel Running Wheel Mouse Model for Botulism and its use for the Evaluation of Post-Symptom Antitoxin Efficacy. Antimicrob. Agents Chemother..

[17] Tacket C.O., Shandera W.X., Mann J.M., Hargrett N.T., Blake P.A. (1984). Equine antitoxin use and other factors that predict outcome in type A foodborne botulism. Am. J. Med..

[18] Sheth A.N., Wiersma P., Atrubin D., Dubey V., Zink D., Skinner G., Doerr F., Juliao P., Gonzalez G., Burnett C. (2008). International outbreak of severe botulism with prolonged toxemia caused by commercial carrot juice. Clin. Infect. Dis..

[19] Diamant E., Pass A., Rosen O., Ben David A., Torgeman A., Barnea A., Tal A., Rosner A., Zichel R. (2018). A Novel Rabbit Spirometry Model of Type E Botulism and Its Use for the Evaluation of Postsymptom Antitoxin Efficacy. Antimicrob. Agents Chemother..

[20] Torgeman A., Schwartz A., Diamant E., Baruchi T., Dor E., Ben David A., Pass A., Barnea A., Tal A., Rosner A. (2018). Studying the differential efficacy of postsymptom antitoxin treatment in type A versus type B botulism using a rabbit spirometry model. Dis. Model. Mech..

[21] Vazquez-Cintron E., Machamer J., Ondeck C., Pagarigan K., Winner B., Bodner P., Kelly K., Pennington M.R., McNutt P. (2020). Symptomatic treatment of botulism with a clinically approved small molecule. JCI Insight.

[22] U.S. Food and Drug Administration Product Development under the Animal Rule Guidance for Industry. https://www.fda.gov/media/88625/download.

[23] Hedenqvist P., Edner A., Fahlman A., Jensen-Waern M. (2013). Continuous intravenous anaesthesia with sufentanil and midazolam in medetomidine premedicated New Zealand White rabbits. BMC Vet. Res..

[24] Weber K., Mowat V., Hartmann E., Razinger T., Chevalier H.J., Blumbach K., Green O.P., Kaiser S., Corney S., Jackson A. (2011). Pathology in Continuous Infusion Studies in Rodents and Non-Rodents and ITO (Infusion Technology Organisation)-Recommended Protocol for Tissue Sampling and Terminology for Procedure-Related Lesions. J. Toxicol. Pathol..

[25] Kamaruzaman N.A., Kardia E., Kamaldin N., Latahir A.Z., Yahaya B.H. (2013). The rabbit as a model for studying lung disease and stem cell therapy. BioMed Res. Int..

[26] Shiomi M., Houdebine L.-M., Fan J. (2009). Rabbit as a Model for the Study of Human Diseases. Rabbit Biotechnology—Rabbit Genomics, Transgenesis, Cloning and Models.

[27] Jacobson A.R., Adler M., Silvaggi N.R., Allen K.N., Smith G.M., Fredenburg R.A., Stein R.L., Park J.B., Feng X., Shoemaker C.B. (2017). Small molecule metalloprotease inhibitor with in vitro, ex vivo and in vivo efficacy against botulinum neurotoxin serotype A. Toxicon.

[28] McNutt P.M., Vazquez-Cintron E.J., Tenezaca L., Ondeck C.A., Kelly K.E., Mangkhalakhili M., Machamer J.B., Angeles C.A., Glotfelty E.J., Cika J. (2021). Neuronal delivery of antibodies has therapeutic effects in animal models of botulism. Sci. Transl. Med..

[29] Miyashita S.I., Zhang J., Zhang S., Shoemaker C.B., Dong M. (2021). Delivery of single-domain antibodies into neurons using a chimeric toxin-based platform is therapeutic in mouse models of botulism. Sci. Transl. Med..

[30] Pang Y.P., Davis J., Wang S., Park J.G., Nambiar M.P., Schmidt J.J., Millard C.B. (2010). Small molecules showing significant protection of mice against botulinum neurotoxin serotype A. PLoS ONE.

[31] Burnett J.C., Opsenica D., Sriraghavan K., Panchal R.G., Ruthel G., Hermone A.R., Nguyen T.L., Kenny T.A., Lane D.J., McGrath C.F. (2007). A Refined Pharmacophore Identifies Potent 4-Amino-7-chloroquinoline-Based Inhibitors of the Botulinum Neurotoxin Serotype A Metalloprotease. J. Med. Chem..

[32] Eichhorn T., Dolimbek B.Z., Deeg K., Efferth T., Atassi M.Z. (2012). Inhibition in vivo of the activity of botulinum neurotoxin A by small molecules selected by virtual screening. Toxicon.

[33] Eubanks L.M., Hixon M.S., Jin W., Hong S., Clancy C.M., Tepp W.H., Baldwin M.R., Malizio C.J., Goodnough M.C., Barbieri J.T. (2007). An in vitro and in vivo disconnect uncovered through high-throughput identification of botulinum neurotoxin A antagonists. Proc. Natl. Acad. Sci. USA.

[34] Singh P., Singh M.K., Chaudhary D., Chauhan V., Bharadwaj P., Pandey A., Upadhyay N., Dhaked R.K. (2012). Small-molecule quinolinol inhibitor identified provides protection against BoNT/A in mice. PLoS ONE.

[35] Diamant E., Torgeman A., Ozeri E., Zichel R. (2015). Monoclonal Antibody Combinations that Present Synergistic Neutralizing Activity: A Platform for Next-Generation Anti-Toxin Drugs. Toxins.

[36] Li B., Peet N.P., Butler M.M., Burnett J.C., Moir D.T., Bowlin T.L. (2010). Small molecule inhibitors as countermeasures for botulinum neurotoxin intoxication. Molecules.

[37] Mazuet C., Dano J., Popoff M.R., Creminon C., Volland H. (2010). Characterization of botulinum neurotoxin type A neutralizing monoclonal antibodies and influence of their half-lives on therapeutic activity. PLoS ONE.

[38] Nowakowski A., Wang C., Powers D.B., Amersdorfer P., Smith T.J., Montgomery V.A., Sheridan R., Blake R., Smith L.A., Marks J.D. (2002). Potent neutralization of botulinum neurotoxin by recombinant oligoclonal antibody. Proc. Natl. Acad. Sci. USA.

[39] Duplantier A., Kane C., Bavari S. (2016). Searching for Therapeutics against Botulinum Neurotoxins: A True Challenge for Drug Discovery. Curr. Top. Med. Chem..

[40] Dor E., David T., Dekel Jaoui H., Schwartz A., Baruchi T., Torgeman A., Ben David A., Rosen O., Tal A., Rosner A. (2021). A Rabbit Model for Prolonged Continuous Intravenous Infusion via a Peripherally Inserted Central Catheter. Front. Pharmacol..

[41] Binz T., Kurazono H., Wille M., Frevert J., Wernars K., Niemann H. (1990). The complete sequence of botulinum neurotoxin type A and comparison with other clostridial neurotoxins. J. Biol. Chem..

[42] GenBank: M30196.1 C.botulinum Neurotoxin Gene, Complete Cds. https://www.ncbi.nlm.nih.gov/nuccore/M30196.

[43] Diamant E., Lachmi B.E., Keren A., Barnea A., Marcus H., Cohen S., David A.B., Zichel R. (2014). Evaluating the synergistic neutralizing effect of anti-botulinum oligoclonal antibody preparations. PLoS ONE.

[44] Torgeman A., Mador N., Dorozko M., Lifshitz A., Eschar N., White M.D., Wolf D.G., Epstein E. (2017). Efficacy of inactivation of viral contaminants in hyperimmune horse plasma against botulinum toxin by low pH alone and combined with pepsin digestion. Biologicals.

[45] European Directorate for the Quality of Medicines and Healthcare (2019). European Pharmacopoeia (Botulinum antitoxin).

